# Case of Scirrhus of the Stomach

**Published:** 1840-02-08

**Authors:** Thomas Miller

**Affiliations:** Professor of Surgery in the Columbian College, Washington


### CASE OF SCIRRHUS OF THE STOMACH. By Thomas Miller, M. D., *Professor of Surgery in the Columbian College, Washington.*

The Rev. William M'Sherry, president of Georgetown College, D. C., a catholic clergyman of distinction, a man of most exemplary habits, kind disposition, and urbane manners, for sixteen years previous to death, complained of a general costive habit, and occasional dyspeptic symptoms and biliary derangements. He dated the inception of his indisposition from an attack of bilious fever, that he laboured under at Turin, in 1823, where he underwent a course of the active purgative treatment of the celebrated Curatif Le Roy. It was here that he first felt an unusual hard lump in the epigastrium, that gave him at the time of his sickness some slight pain, but not sufficient, he said, to create any alarm, or induce him to bring it under the eye of his physician. The pain disappearing entirely upon recovery from the attack, and the tumour seeming to diminish, it attracted his attention no farther, except at the subsequent periods of visceral derangement, that he was liable to, upon any the slightest imprudence, when, he said, it always gave him some slight degree of pain, and seemed somewhat to enlarge. But (as in the first attack) it would subside, and the pain cease, simultaneously upon recovery. His appetite was generally good, and his digestion, though sluggish, seemed unimpaired, except at such intervals of sickness, until the spring of 1839, when thinking that his usual diet disagreed with him, he put himself upon one exclusively vegetable and ate very heartily, and for awhile seemed to digest readily every variety of salad, from that time until the succeeding summer. But his complaint began rapidly to gain ground upon him, and about the commencement of summer he made frequent complaints of indigestion, and thought he could discern some slight pulsation, and an unusual increase in size of the lump at his breast bone, as he expressed it. His countenance became cadaverous, he was troubled with dyspnœa upon slight exertion, and complained of a feeling of sinking in the præcordia, and a fluttering sensation in the part when standing for any length of time in an erect posture. He still kept, however, at his usual routine of duties until the commencement of college vacation in July; when at the advice of a friend he undertook a journey to the lower counties of Maryland, and consulted upon the way a gentleman of professional celebrity at Baltimore, who prescribed for him the use of iodine internally and externally.

He returned home shortly afterwards, and continued for awhile under this treatment, but finding no relief, called upon the attending physician of the college, Dr. Warfield, and for the first time brought the tumour under his observation, and submitted it to his inspection. He was then put upon a mild sedative treatment—abstinence and rest. The palpitation, however, continued unabated, and the tumour increasing. Pressure now, in any considerable degree, produced pain; a hacking cough supervened, accompanied with thick mucous expectoration, dyspnœa increased, and the least change of position produced faintness; and his bowels became extremely torpid, requiring the frequent application of enemata. At this time, about the first of December, Dr. Miller was called in—made a stethoscopic examination of the thorax, and abdomen over the seat of the tumour, which now communicated to the hand a sense of violent pulsation extending from the right to the left hypochondrium. Effusion in the right lung was detected, and though the examination could not then clearly detect the nature of the malady, it demonstrated enough to disprove the impression of any organic derangement of the heart, that his friends were apprehensive he was labouring under.

*December 12th.*—Was examined by Drs. Wright of Baltimore and Gerhard of Philadelphia, in addition to the others in attendance. Symptoms unchanged—but had now for some days been troubled with a frequent vomiting of a substance resembling coffee grounds—elucidating clearly, the prominent pathological derangement; strength continued rapidly to decline until December 17th, when he gradually sunk, calm and recollected, and perfectly sensible of the approach of death, which took place at 12 o'clock, P. M.

*December 18th.*—Autopsy twenty hours after death.

In the presence of Drs. P. Warfield, N. W. Worthington, and J. A. Ritchie. Examined his chest and abdomen.

*Thorax.*—Externally slightly distended, more on the left than on the right side. On raising the sternum, it was discovered that numerous adhesions, recent and old, existed between the pleuræ on both sides. In the right side more than a quart of serum ; the right lung adhered firmly to the back part of the thorax. Tubercles in different stages were found in the *lower* lobe of the right lung ; the parenchyma of the upper lobe of the lung generally healthy.

*Left Lung.*—Mottled, emphysematous; fewer pleuritic adhesions than in the right: much congested, had the appearance of being double the size of the right, owing to the partial collapse of the right, while the left did not collapse at all. (It should here be remarked, that Mr. M'S. had been occasionally troubled with slight asthma ;) no tubercles in the left lung.

*Heart.*—Pericardium contained about ℥iv of serum ; heart healthy.

*Abdomen.*—Peritoneum about the epigastric region thickened and studded with lymph. Considerable serous effusion in the cavity of peritoneum.

*Stomach.*—This organ extended from the liver to the spleen, adhered firmly to the diaphragm and liver above, to the pancreas posteriorly, to the duodenum, liver and spleen laterally, and to the colon and parts below. It was hard, firm, and greatly thickened ; when removed and opened, it was found to contain about one-half pint of the same coffee-ground substance which he had been ejecting for some days before death. About four-fifths of it was converted into a dense scirrhous mass : the pyloric orifice was scarcely large enough to admit a large sized bougie as far as the junction with the duodenum ; it was thickened to one and a half inches, and this character gradually extended, until about four-fifths of the stomach was involved ; the remaining one-fifth, that is, the left extremity including the cardiac orifice, was free from the disease. The three coats of the stomach could be distinctly perceived in the thickened mass ; the greatest degree of thickening seemed to be in the muscular coat ; the serous was least affected, the mucous was thickened and much injected. In many points the vessels were distinctly seen, and the rugæ, particularly about the pylorus, much enlarged, and some ulcerous patches. The only part of the stomach that appeared at all capable of carrying on digestion, was a small portion embracing a circumference of not more than two inches at the left extremity of the great cul de sac.

*Liver*, much enlarged, tissue friable, much congested ; peritoneal coat easily removed.

*Gall bladder* filled with healthy bile.

*Intestines.*—All gave evidence of having been much inflamed ; the duodenum and jejunum were in a sphacelated state ; when handled, they gave way readily, and contained a quantity of the same substance as the stomach. The evidences of disease in the large intestines were more recent ; they were filled with scyballa and flatus.

The *pancreas* was much enlarged and softened, as also the mesenteric glands. The mesentery was very much elongated, so much so as to admit of the small intestines resting on the thighs when the abdomen was opened.

The arteries springing from the abdominal aorta were all greatly enlarged, as had been anticipated.

This may be considered a very remarkable case ; the person had evidently been labouring under the disease for sixteen years previous to his death, and during the time the only complaint he experienced was costiveness, slight dyspepsia, and occasional functional hepatic derangements. He attended to his vocation to within a few weeks of his death, and up to that time enjoyed tolerable health, or, if he suffered pain, was never heard to complain. And still more strange is it, to perceive how long life may be sustained by a very small portion of healthy stomach ; only a few inches of *tolerably* healthy mucous membrane existed, for it was not, at the time of his death, truly healthy. How long *this* extent of disease had existed, it is impossible to ascertain, but if we were allowed to surmise from its character, we should say that for many months, if not for a year or two, not more than one-fifth of his stomach had been supplying healthy secretion and healthy chyle for the support of his system. For in this case death was evidently produced by starvation, from inability of the stomach to contain and assimilate sufficient injesta.

If he could have lived a few months more, he would have experienced, in all probability, an increase of pain, and we should have found ulcers. This we infer from what we have seen in other cases.

